# Polyneuropathy, Organomegaly, Endocrinopathy, Monoclonal Gammopathy, and Skin Changes (POEMS) Syndrome With IgG Kappa/IgG Lambda Biclonal Gammopathy: A Rare Presentation of a Rare Disease

**DOI:** 10.7759/cureus.10232

**Published:** 2020-09-03

**Authors:** Leonardo Mejia Buritica, Vanessa Santiago-Pacheco, Oliver Perilla, Dayana Quintero

**Affiliations:** 1 Department of Hematology, Universidad de Antioquia, Medellín, COL; 2 Department of Hematopathology, Universidad de Antioquia, Medellín, COL; 3 Department of Internal Medicine, Universidad Pontificia Bolivariana, Medellín, COL

**Keywords:** poems syndrome, biclonal gammopathy, castleman disease

## Abstract

Polyneuropathy, organomegaly, endocrinopathy, monoclonal gammopathy, and skin changes (POEMS) syndrome is a low prevalence multisystemic paraneoplastic disease. The name is an acronym composed by its most relevant clinical manifestations, which are polyneuropathy, organomegaly, endocrinopathy, monoclonal gammopathy, and skin changes. More than 95% of the POEMS syndrome cases are monoclonal for lambda light chains; however, few cases have been reported in the literature with a biclonal component. In this paper, we report a rare case of a patient who has POEMS syndrome with biclonal gammopathy. To the best of our knowledge, this is the first reported case in the literature of POEMS syndrome with expression of IgG kappa/IgG lambda biclonal gammopathy.

## Introduction

Polyneuropathy, organomegaly, endocrinopathy, monoclonal gammopathy, and skin changes (POEMS) syndrome is a low prevalence multisystemic paraneoplastic disease. The name is an acronym that highlights the most significant features: polyneuropathy, organomegaly, endocrinopathy, monoclonal gammopathy, and skin changes [1]. Other important manifestations of POEMS include fever, papilledema, volume overload, sclerotic bone lesions, thrombocytosis, erythrocytosis, and elevated levels of vascular endothelial growth factor (VEGF) [2]. Other names have previously been used to refer to the same clinical condition, such as Crow-Fukase syndrome, osteosclerotic myeloma, and Takatsuki syndrome [3]; however, the term POEMS introduced in 1980 is the most widely used nowadays [4].

It has been reported that between 11% and 30% of patients with POEMS syndrome have Castleman disease (giant lymph node hyperplasia) [5]; nevertheless, this percentage may be underestimated since not all patients undergo lymph node biopsy. A group of patients with Castleman disease without evidence of plasma cell neoplasm or neuropathy (and clinical characteristics of POEMS syndrome) has been described. These patients should be more accurately referred to as the Castleman disease variant of POEMS syndrome, since they may sometimes have a different clinical presentation from classic POEMS syndrome [1].

Regarding the monoclonal protein, more than 95% of the POEMS syndrome cases are monoclonal for lambda light chains [1]; however, few cases have been reported in the literature with a IgG kappa/IgA lambda biclonal component [6]. To the best of our knowledge, this article presents the first clinical case of a patient with POEMS syndrome with expression of IgG kappa/IgG lambda biclonal gammopathy.

## Case presentation

A 67-year-old woman with a history of hypertension and dyslipidemia presented with a five-month history of loss of strength in upper and lower extremities, progressive until bedridden. She also reported weight loss, fatigue, and generalized bone pain.

Physical examination revealed generalized hyperpigmentation and white nails, bilateral axillary lymphadenopathies of 3 cm in diameter, non-painful hepatomegaly, and pitting edema of the lower limbs. Neurological examination showed predominantly distal weakness, generalized areflexia, hypoesthesia of the legs, and inability to walk.

The complete blood count (CBC) revealed hemoglobin 16.5 g/dL, hematocrit 48.6%, white blood cell count 10,200 cells/µL, and a normal platelet count 423,000/µL. Endocrinological studies revealed mild hyperparathyroidism with parathyroid hormone (PTH) 72.9 pg/mL (reference range 15-68.3 pg/mL), primary hypothyroidism with thyroid-stimulating hormone (TSH) 30.6 µIU/mL (reference range 0.55-4.78 µIU/mL), and free thyroxine (FT4) 0.31 ng/dL (reference range 0.89-1.76 ng/dL). Liver and kidney tests were within the reference range. Studies for HIV, hepatitis B, hepatitis C, and syphilis were all negative.

**Figure 1:**
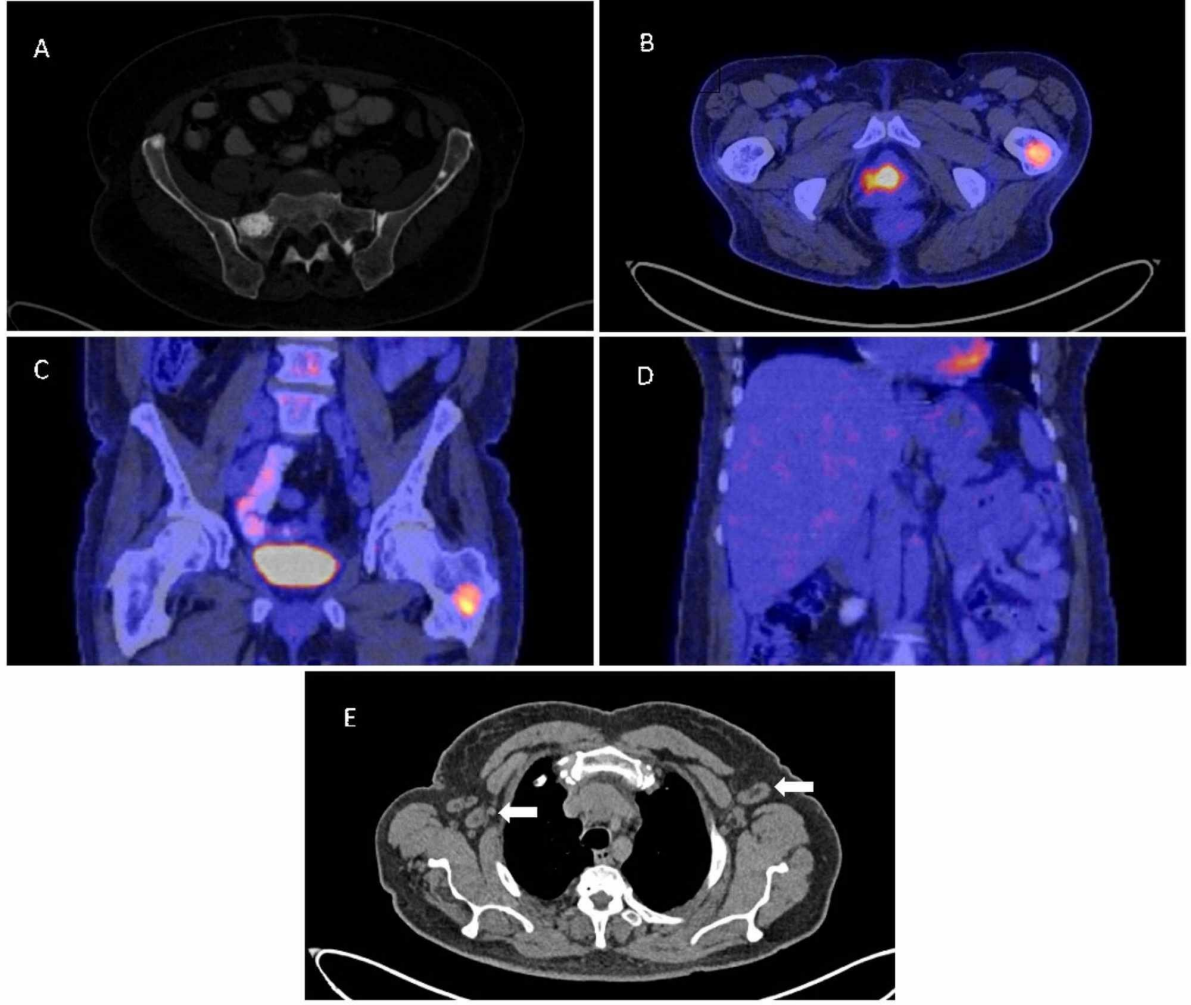
Positron emission tomography CT (PET/CT) findings (A) Sclerotic lesions in the sacrum and iliac bone in cross section. (B) Sclerotic lesion with metabolic activity in the left femur in cross section. (C) Sclerotic lesion with metabolic activity in the left femur in coronal section. (D) Hepatomegaly in coronal section. (E) Bilateral axillary adenomegalies (arrows) in cross section.

Four-limb electromyography was performed, which showed demyelinating polyneuropathy of the lower extremities. Positron emission tomography CT (PET/CT) revealed hepatomegaly and polyostotic sclerotical bone lesions in both the right and the left humerus, the sternum, L3 vertebral body, the right acetabulum, the left sphenoid, the sacrum, and the trochanteric region of left femur (Figure 1).

**Figure 2:**
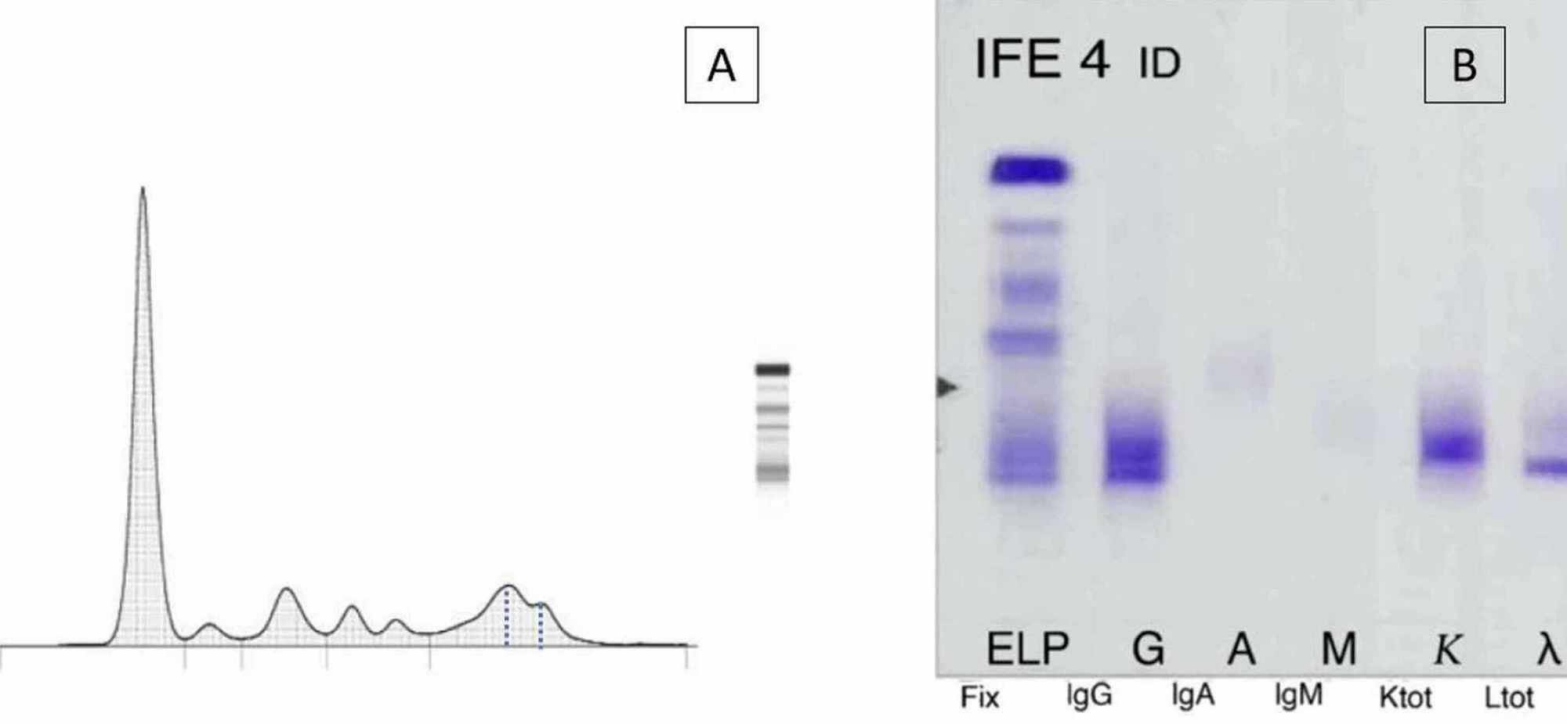
Serum electrophoresis and immunofixation (A) Protein electrophoresis. (B) Serum immunofixation

Double peak in gamma was observed in serum protein electrophoresis and a biclonal component with IgG kappa/IgG lambda was demonstrated on immunofixation (Figure 2).

**Figure 3:**
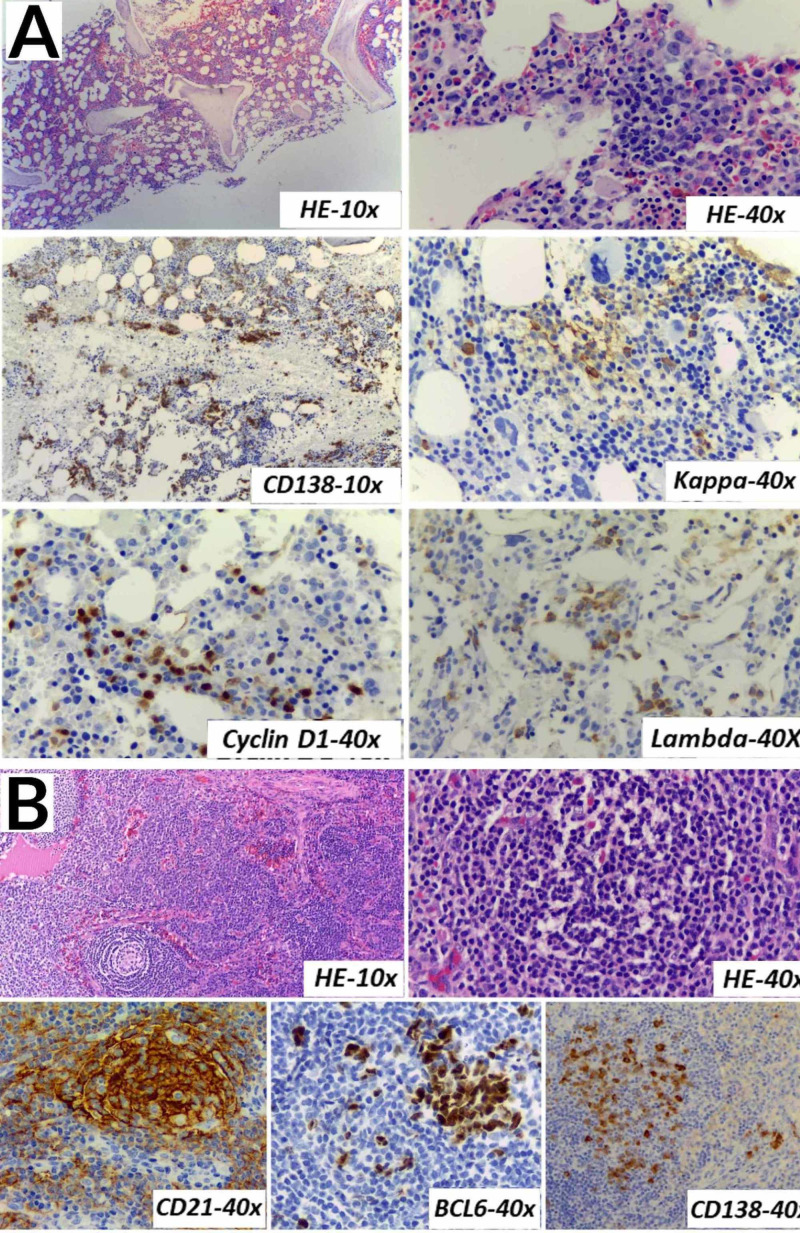
Bone marrow and cervical lymph node (A) Bone marrow: It shows an increase in plasma cells with small aggregates, occupying approximately 10% of the medullary spaces (CD138), with preserved kappa/lambda expression and aberrant expression of cyclin D1. (B) Cervical lymph node: Lymphoid follicles with concentric hyperplasia of the mantle zone, proliferation of follicular dendritic cells (CD21), and small depleted germinal centers (BCL6) are observed. There is a low to moderate number of plasma cells (CD138). HE*:* hematoxylin and eosin stain, CD:* *cluster of differentiation, BCL6*: *B-cell lymphoma 6 protein.

The light chain ratio was normal. Bone marrow biopsy revealed an increase in plasma cells with small aggregates, occupying approximately 10% of the medullary spaces with CD138 expression, with preserved kappa/lambda expression and aberrant expression of cyclin D1, which is associated with the 11;14 translocation. The biopsy of a cervical lymph node revealed morphological and immunophenotypic findings compatible with Castleman disease (Figure 3).

Treatment with lenalidomide and dexamethasone was started, with a rapid improvement in neurological symptoms.

## Discussion

POEMS is a rare paraneoplastic clinical syndrome secondary to the presence of a plasma cell neoplasm [1]. The pathophysiology is not well understood, but it has been related to an increase in inflammatory cytokines, mainly VEGF [7]. Diagnostic criteria have been proposed by the International Myeloma Working Group [8].

Our case had biclonal component with IgG/kappa and IgG/lambda expression, which is rare in patients with POEMS, who usually have a lambda light chain monoclonal component in most cases [1]. Biclonal gammopathy is defined by the presence of two monoclonal components, which can be produced by the presence of two different plasma cell clones or by a single clone producing two monoclonal chains [6].

Biclonal gammopathy is rare and has been reported in 2.5% of cases of patients with monoclonal protein, most with a diagnosis of monoclonal gammopathy of undetermined significance in 65% of cases and multiple myeloma in 16% of cases [9]. Regarding the biclonal component, the most frequent finding is the combination of IgG and IgA observed in 53% of cases, followed by the combination of IgM and IgG in 26% of cases [10]. Biclonal gammopathy with the combination of the IgG heavy chain with both monoclonal kappa and lambda light chains has been reported in non-Hodgkin lymphoma [11]. In POEMS syndrome, there are very few reported cases of biclonal gammopathy; the reports by De et al. [6] and Ham et al. [12] correspond to the combination of IgG and IgA. To our knowledge, this is the first reported case in the literature of POEMS syndrome with expression of IgG kappa/IgG lambda biclonal gammopathy.

Castleman disease, also known as angiofollicular lymph node hyperplasia, is a rare lymphoproliferative disease, which has been reported in up to 30% of patients with POEMS [5]. The classic POEMS syndrome must be differentiated from the Castleman disease variant of the POEMS syndrome [13]. In classic POEMS syndrome, there is evidence of plasma cell neoplasia and monoclonal component, with demyelinating peripheral neuropathy; the finding of Castleman disease in the lymph nodes may also be present. In contrast, in the Castleman disease variant of POEMS syndrome, there is no evidence of plasma cell neoplasia or monoclonal component and peripheral neuropathy is rare. In these cases, the presence of Castleman disease is necessary to establish the diagnosis [8]. Our case meets criteria for classic POEMS syndrome due to the presence of demyelinating polyneuropathy, monoclonal component, sclerotic bone lesions, enlarged lymph nodes, hepatomegaly, edema, endocrinopathy, and hyperpigmentation. The finding of Castleman's disease, in this case, additionally supports the diagnosis of POEMS syndrome.

The treatment of classic POEMS syndrome is usually directed at the plasma cell clone and depends mainly on whether there is a presence of solitary plasmacytoma or plasma cell infiltration in the bone marrow biopsy. For patients with isolated plasmacytoma without infiltration of plasma cells into the bone marrow, radiation therapy directed to plasmacytoma is the treatment of choice, which can resolve the clinical manifestations of the disease with an overall survival of 97% and failure-free survival of 52% at four years of follow-up [14].

Patients with two or more plasmacytomas or infiltration by plasma cells in the bone marrow can no longer be cured with radiotherapy and require systemic therapy, like that used in patients with multiple myeloma. Schemes combining bortezomib, cyclophosphamide, and dexamethasone (VRd) [15] or lenalidamide and dexamethasone (Rd) [16,17] have been reported with good results.

In the case of young patients with multiple sclerotic lesions and severe clinical manifestations, high doses of melphalan chemotherapy followed by autologous stem cell transplantation should be considered. Significant improvement has been demonstrated with this therapy, especially in peripheral neuropathy [18].

## Conclusions

POEMS syndrome is a rare disease associated with a plasma cell neoplasm. Its clinical manifestations are diverse, and the diagnosis is usually delayed. The classic form of the disease presents with demyelinating peripheral neuropathy and monoclonal gammopathy, usually lambda light chain. We present the case of a patient with IgG kappa/IgG lambda biclonal gammopathy associated with Castleman disease, who meets criteria for classic POEMS syndrome. Biclonal gammopathy is an exceedingly rare finding in POEMS syndrome. The importance of this finding regarding clinical evolution and response to treatment has not yet been established. With this case, we hope that the diagnosis of POEMS syndrome will also be considered in patients with this presentation.
